# A Comparative Analysis of Nursing Care Needs Scores at Discharge and Transfer Among Survivors of Critical COVID-19 and Septic Shock: A Retrospective Observational Study Using Modified Poisson Regression From Japan

**DOI:** 10.7759/cureus.74032

**Published:** 2024-11-19

**Authors:** Jun Kawabata, Kotaro Kuwaki

**Affiliations:** 1 Advanced Emergency Medical Service Center, Kurume University Hospital, Kurume, JPN; 2 Public Health, Kurume University, Kurume, JPN

**Keywords:** covid-19, critical care and hospital medicine, inpatient data, nursing analytics, nursing care needs, post-intensive care syndrome (pics), septic shock, ventilator use

## Abstract

Background

Research on nursing care needs (NCNs) for critically ill patients at discharge is scarce. This study aimed to quantify and compare NCNs at discharge between patients with severe COVID-19 and septic shock and to identify factors associated with higher NCNs.

Methodology

We retrospectively analyzed data from the Diagnosis Procedure Combination database between April 1, 2020, and March 31, 2023, on patients requiring ventilators in the intensive care unit (ICU). We excluded patients who were <15 years old, died during hospitalization, had multiple admissions during the study period, had incomplete medical records, and received extracorporeal membrane oxygenation. Differences in patient condition and nursing care implementation between COVID-19 and septic shock patients were compared using the Mann-Whitney U test. A modified Poisson regression model was used to assess factors associated with NCNs scores of 7 or more. Covariates included continuous variables such as age, duration of ventilation, length of stay, sex, use of sleeping medications, use of delirium medications, presence of risky behavior at admission, use of continuous hemofiltration, and comorbidities including cerebrovascular diseases. A categorical variable classified patients into the following three groups: COVID-19, septic shock, or other diseases.

Results

Among 438 ventilated patients, 33 had COVID-19, and 37 had septic shock. The Mann-Whitney U test showed no significant differences in patient condition (8 vs. 8, p = 0.34) or nursing care implementation (4.0 vs. 4.0, p = 0.72). Multivariable analysis revealed that COVID-19 was associated with a slightly higher NCNs score of ≥7 (risk ratio (RR) = 1.42, 95% confidence interval (CI) = 1.06-1.89, p = 0.018), older age (RR = 1.02, 95% CI = 1.00-1.02, p < 0.01), and prolonged ventilation (RR = 1.02, 95% CI = 1.01-1.02, p < 0.01). Notably, the use of sleeping medications was associated with a lower NCNs score (RR = 0.68, 95% CI = 0.57-0.83, p < 0.01).

Conclusions

While no statistically significant differences in NCNs were found between the COVID-19 and sepsis survivor groups, those groups demonstrated higher NCNs levels upon discharge. These findings could help expedite ICU liberation for critical survivors and provide valuable insights to inform evidence-based nursing practice. Our findings suggested that NCNs use may enhance the quality of nursing care, promote further nursing research, and contribute valuable insights to critical care nursing and post-ICU patient management.

## Introduction

Patients admitted to the intensive care unit (ICU) often face life-threatening conditions, necessitating highly invasive treatments and sophisticated care management. A well-documented phenomenon known as post-intensive care syndrome (PICS) has been observed in these critically ill patients. PICS can manifest during the ICU stay, persist after ICU discharge, and continue following hospital release [1-3]. PICS not only affects patients’ long-term prognosis but also impacts the mental well-being of their families and poses significant social and economic burdens on healthcare systems [4].

Multiple studies have reported on the post-ICU sequelae in COVID-19 patients [5-7], who often exhibit a high degree of dependence in activities of daily living (ADL) at discharge [8]. Similarly, septic shock patients frequently experience PICS-related impairments [9] and demonstrate reduced quality of life at discharge [10]. These critically ill patients require sustained support for ADLs during hospitalization and post-discharge, suggesting a persistently high level of nursing care needs (NCNs). We hypothesize that COVID-19 patients requiring mechanical ventilation may require higher NCNs compared to septic shock patients.

The Nursing Activities Score (NAS) has been used to evaluate nursing workload, demonstrating high care needs for patients with COVID-19 and septic shock [11,12]. However, research quantitatively examining NCNs at discharge or transfer remains scarce. In the context of increasing emphasis on mitigating PICS from ICU admission [13], a quantitative evaluation of nursing care at discharge from acute care is crucial. This assessment can serve as a benchmark for graduated nursing care during patients’ transition through recovery stages, from hospital discharge to rehabilitation, chronic care, and, ultimately, home care.

In Japan, the Diagnosis Procedure Combination (DPC) system offers valuable data for analyzing and optimizing healthcare expenditures while improving medical care quality [14]. This comprehensive dataset includes measuring the intensity of the NCNs index. These databases enable quantitative evaluation of nursing care about patients’ daily conditions and the level of support required for ADLs. Despite the availability of quantitative data on NCNs in Japan, research using this data for comprehensive analysis is lacking. To address this gap, our study aims to examine the differences in NCNs at discharge between COVID-19 and septic shock patients requiring mechanical ventilation, as well as the factors associated with higher NCNs.

This study will contribute valuable insights to critical care nursing and post-ICU patient management. The findings provide information for discharge planning, resource allocation, and the development of targeted interventions for these high-acuity patient populations. Moreover, this quantitative research using NCNs data in Japan represents a significant advancement in the field of nursing science, with the potential to yield multifaceted benefits for both national and international nursing practices.

## Materials and methods

Study design

This retrospective observational study was conducted at a single tertiary acute care hospital serving a population of approximately 300,000 residents. The hospital is designated as a DPC institution, providing advanced medical care, developing innovative medical technologies, and offering training in advanced medicine to support the regional healthcare system.

Data source

This retrospective study used the DPC database between April 1, 2020, and March 31, 2023. The DPC system aims to optimize healthcare expenditures and standardize medical care nationwide by aggregating medical claim data. For this analysis, medical claim cases were extracted from the DPC database and linked using patient identification numbers. Three datasets were (1) Form 1 (FF1), containing patient summary information; (2) the EF file, detailing inpatient medical claims; and (3) the H file, including NCNs score [14]. The patient summary data included admission and discharge dates, sex, age, comorbidities (International Classification of Diseases, Tenth Revision (ICD-10)), height, weight, and ADLs at admission. The medical claim data included details on surgeries, diagnostic tests, injections, medications, medical fee incentives, and so forth. NCNs data were derived from daily records completed by nurses. This tool evaluated critical treatments, management, and nursing care required each day, serving as an indicator of nursing care quality and enabling appropriate allocation of nursing resources. Although NCNs data were included in both Section A (monitoring, procedures, etc.) and Section B (patient condition and care implementation), this present study focused on Section B, which assesses patients’ daily living activities and provides a standardized evaluation across ICU and general ward settings. Section B is a composite index consisting of 11 items that evaluate patient conditions and nursing care implementation. The patients’ condition was evaluated based on the following five items: turning in bed, transferring, eating, dressing, and oral hygiene. Each item was scored on a three-point scale, with 0 points indicating full independence, 1 point indicating partial assistance, and 2 points indicating total assistance. For oral hygiene, 0 points indicated full independence, while 1 point indicated the need for assistance. The maximum achievable score for patient condition assessment was 9 points, and the minimum was 0 points. Nursing care implementation was assessed using the remaining four items, excluding turning in bed. Each item was scored as 0 points if no assistance was required and 1 point if assistance was required. The maximum achievable score for this component was 4 points, and the minimum was 0 points. In addition to patient conditions and NCNs data, Section B also assessed patient compliance with inpatient care, adherence to medical orders, and the presence of risky behavior. The overall Section B score was calculated by summing the scores of all 11 items. Due to the unavailability of data on the need for care on the day of discharge or transfer, the score from the previous day was used as the final evaluation (Appendices).

Inclusion and exclusion criteria

This retrospective cohort study included adult patients (≥15 years) admitted to the ICU between April 1, 2020, and March 31, 2023. Patients were eligible if they required mechanical ventilation (medical fee codes J045 and J040). Primary diagnoses of COVID-19 (ICD-10: U071) and septic shock (ICD-10: A41.9) were identified based on the most resource-intensive condition documented in the patient summary from FF1 data. Inclusion criteria required that patients who experienced ICU care survived to discharge or transfer from the hospital. We excluded patients who were <15 years old, died during hospitalization, had multiple admissions during the study period, had incomplete medical records, and received extracorporeal membrane oxygenation.

Ethical statement

The study was approved by the Kurume University Ethics Committee (approval number: 22229) on March 7, 2023, and subsequently authorized by the hospital director on March 9, 2023. All data were anonymized to prevent the identification of personal information, including names and addresses of participants. We opted out of disclosing any identifying information.

Outcomes

The primary outcome was the difference in total scores for patient condition and care implementation items on the NCNs score between the COVID-19 and septic shock groups at the time of hospital transfer or discharge. Secondary outcomes included factors associated with the total NCNs score at discharge, categorized as 1 for scores above the median and 0 for scores below the median.

Statistical analyses

Patient characteristics were described using the mean, median, and interquartile range (IQR) for continuous variables and number and percentage (%) for categorical variables. For the primary outcome, differences in patient condition and care implementation scores were compared using the Mann-Whitney U test. For the secondary outcome, a modified Poisson regression model [15] was used to examine the association between the proportion of patients with a total NCNs score of 7 or more in Section B (a binary variable) and covariates before one day of discharge or transfer. We used a modified Poisson regression model to estimate risk ratios (RRs) and robust standard errors, as this method provides unbiased estimates of RR, is particularly suitable for analyzing common events, and can directly estimate relative risks. Robust regression was applied to mitigate the impact of outliers. The results of the modified Poisson regression are presented as RR with a corresponding 95% confidence interval (CI) and robust standard errors. Although logistic regression was considered, the high proportion of events in the outcome variable posed potential convergence issues, so a modified Poisson regression model was adopted instead. Statistical significance was set at p-values <0.01, and all analyses were performed using R statistical software version 4.1.3 (R Foundation for Statistical Computing, Vienna, Austria).

Covariates

Covariates included continuous variables such as age at admission, duration of mechanical ventilation, and length of stay, along with binary variables indicating sex, use of sleeping medications, use of delirium medications, presence of risky behavior (such as agitated behavior) at admission, use of continuous hemofiltration, and comorbidities including cerebrovascular diseases. A categorical variable classified patients into the following three groups: COVID-19, septic shock, or other diseases. Several studies have reported complications and interventions associated with delirium in hospitalized patients [16,17]. Given the potential impact on nursing care, delirium was defined as the prescription of antipsychotics during hospitalization, and data were extracted on the use of haloperidol (Serenace) and quetiapine (Seroquel) [18,19]. Due to the established association between sleep deprivation and delirium [20], patients prescribed sleep medications at least once during hospitalization were included. Data on the administration of the following sleep medications were extracted: ramelteon (Rozerem), suvorexant (Belsomra), eszopiclone (Lunesta), and lemborexant (Dayvigo) [21,22]. Additionally, to identify patients with delirium, we examined risky behavior documented in NCNs. The presence of such behaviors was considered a potential indicator of delirium, including self-removal of tubes, intravenous lines, falls, and self-harm, and we hypothesized to influence NCNs at discharge.

## Results

Patient characteristics

**Figure 1:**
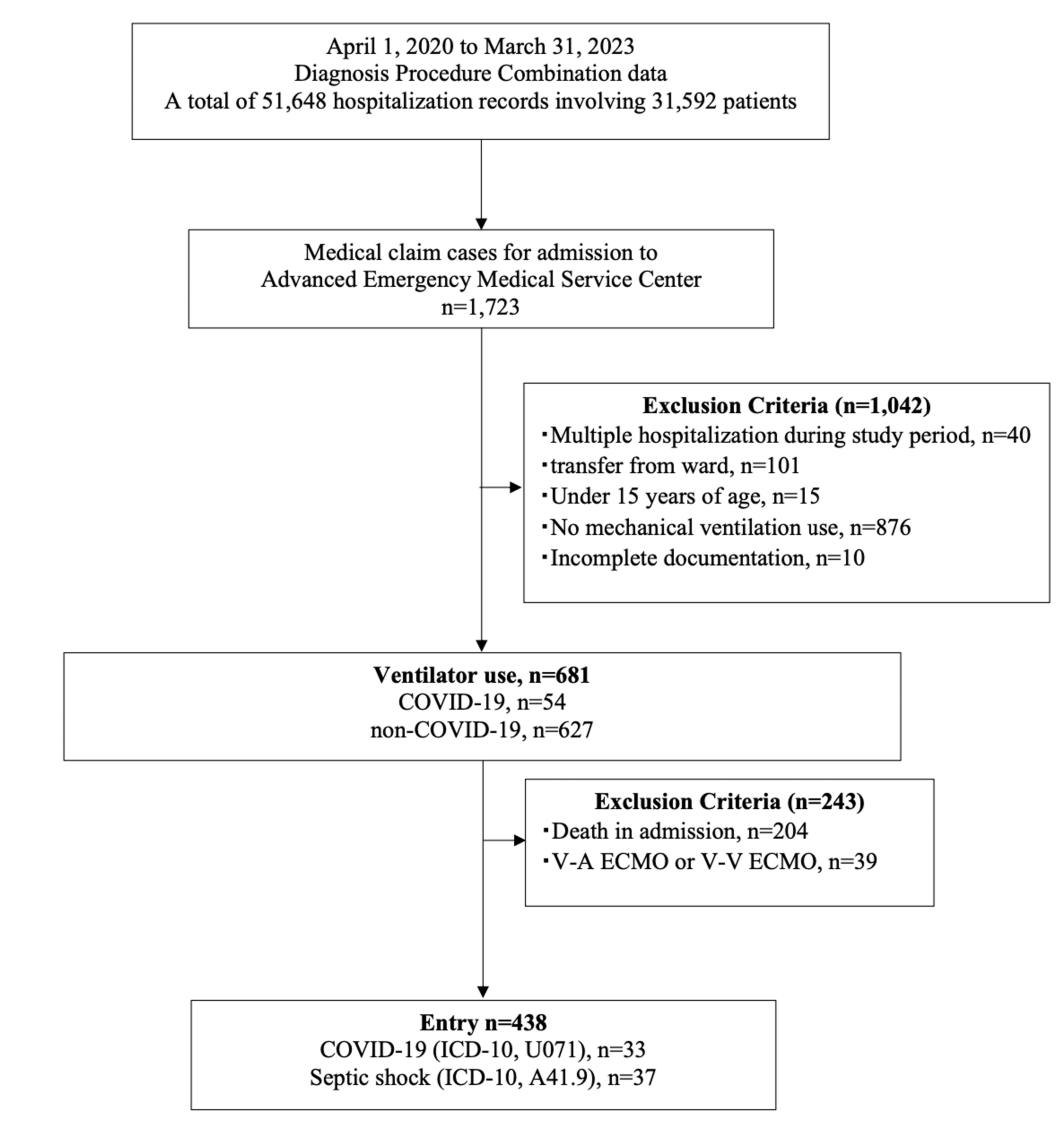
Flowchart for study entry.

Figure 1 shows the patient selection process for this study.

Of the 438 patients admitted to the tertiary care center requiring mechanical ventilation, 33 had COVID-19 and 37 had septic shock after applying exclusion criteria. Table 1 shows the demographic and clinical characteristics of the study population.

**Table 1:** Characteristics of the patients at baseline. IQR = Interquartile range; COVID-19 = coronavirus disease; CHDF = continuous hemodialysis and filtration; SMD = Standardized mean difference.

Characteristics	Others (N=368)	COVID-19 (N=33)	Septic shock (N=37)	SMD COVID-19 vs. septic shock
Female sex, n (%)	148 (40.0)	7 (21.0)	18(49.0)	0.6
Age at admission, median (IQR), years	70 (55, 79)	61 (53, 71)	75 (64, 84)	0.81
Length of stay, median (IQR), days	28(16, 43)	30 (22, 49)	32 (24, 44)	0.16
Ventilator use, median (IQR), days	5 (2, 12)	14 (12, 31)	13 (8, 26)	0.22
CHDF, n (%)	18 (4.9)	3 (9.1)	19 (51)	1.04
Nursing care needs score at discharge, median (IQR), score	6 (2, 9)	7 (5, 9)	7 (4, 9)	0.05
Nursing care needs score at discharge more than 7points, n (%)	173 (47.0)	21 (63.6)	23 (62.2)	0.03
Nursing care needs score at discharge more than 3points, n (%)	269 (73.1)	27 (81.8)	31 (83.8)	0.05
Sleeping medications, n (%)	243 (66.0)	30 (90.9)	31 (83.8)	0.22
Delirium (antipsychotic) medications, n (%)	98 (26.6)	19 (57.6)	10 (27.0)	0.65
Presence of risky behavior, n (%)	131 (35.6)	11 (33.3)	17 (45.9)	0.26
Comorbidity at admission: cerebrovascular disease, n (%)	44 (12.0)	0	1 (2.7)	0.24

The median age at admission was 61 years (53-71 years) for COVID-19 patients and 75 years (64-84 years) for septic shock patients. The median length of stay was 30 days (22-49 days) for COVID-19 patients and 32 days (24-44 days) for septic shock patients. The median duration of mechanical ventilation was 14 days (12-31 days) for COVID-19 patients and 13 days (8-26 days) for septic shock patients. The median NCNs score at discharge was 7 (5-9) for both groups, with 64% of COVID-19 patients and 62% (23/37) of septic shock patients scoring 7 or higher. The prescription rates for sleep and antipsychotic medications were 91% (30/33) and 58% (19/33) for COVID-19 patients, and 84% (31/37) and 27% (10/37) for septic shock patients, respectively.

**Figure 2:**
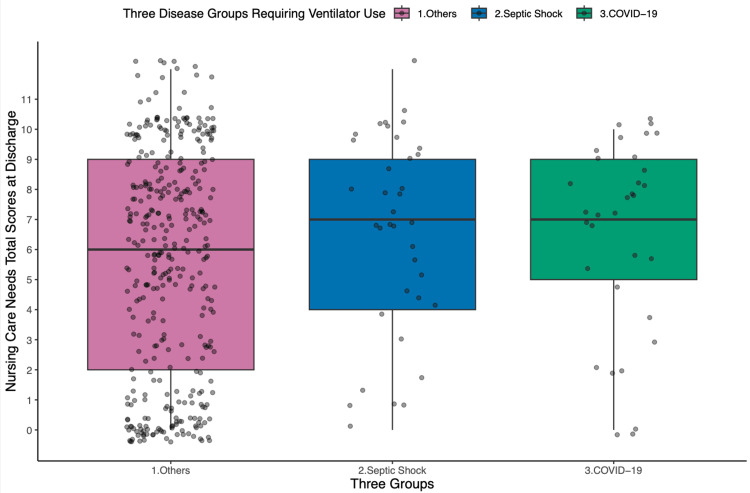
Boxplot of total nursing care needs scores at discharge across three groups.

A binary variable was created using a score of 7 as the cutoff for Poisson regression analysis, as both groups had a median NCNs score of 7 at discharge (Figure 2). According to the 2022 revision of the Japanese medical fee schedule, a total NCNs score of 3 or higher is required for evaluating the acute general inpatient basic fee. Among ICU survivors, 82% of COVID-19 patients and 84% of septic shock patients had a total NCNs score of 3 or higher at discharge.

Outcomes

Table 2 shows the findings for the primary outcome.

**Table 2:** Primary outcome. IQR = interquartile range

Characteristic	COVID-19 (N = 33)	Septic shock (N = 37)	P-value	test
Nursing care needs score at discharge (Patient condition, sum of 5 items), Median (IQR)–score	8 (4, 8)	8 (4, 9)	0.34	Mann–Whitney U test
Nursing care needs score at discharge (Care implementation, sum of 4 items), Median (IQR)–score	4 (3, 4)	4 (3, 4)	0.72	Mann–Whitney U test

There were no statistically significant differences in median pre-discharge NCNs scores for patient condition (8 vs. 8, p = 0.34) and nursing care implementation (4.0 vs. 4.0, p = 0.72) between COVID-19 patients and septic shock patients. Table 3 shows the results of the modified Poisson regression analysis for secondary outcomes in 438 patients.

**Table 3:** Results of the modified Poisson regression analysis. RR = risk ratio; CI = confidential interval; COVID-19 = coronavirus disease; CHDF = continuous hemodialysis and filtration

Covariates	RR	95%CI	P-value
(Intercept)	0.19	0.12–0.31	<0.01
Sepsis (Ref. others)	1.21	0.90–1.61	0.2
COVID-19 (Ref. others)	1.42	1.06–1.89	0.018
Age at admission	1.02	1.01–1.02	<0.01
Female (Ref. male）	0.91	0.76–1.08	0.26
Length of Stay	0.99	0.99–1.00	0.046
Delirium (antipsychotic) medications	0.92	0.72–1.17	0.48
Sleeping medications	0.68	0.57–0.83	<0.01
Presence of risky behavior	1.37	1.15–1.63	<0.01
Ventilator days	1.02	1.01–1.02	<0.01
CHDF	0.9	0.68–1.20	0.47
Cerebrovascular disease	1.38	1.14–1.68	<0.01

Factors associated with an NCNs score of 7 or higher at discharge included COVID-19 (RR = 1.42, 95% CI = 1.06-1.89, p = 0.018), older age at admission (RR = 1.02, 95% CI = 1.00-1.02, p < 0.01), risky behavior at admission (RR = 1.37, 95% CI = 1.15-1.63, p < 0.01), duration of mechanical ventilation (RR = 1.02, 95% CI = 1.01-1.02, p < 0.01), and cerebrovascular disease at admission (RR = 1.38, 95% CI = 1.14-1.68, p < 0.01). Prescription of sleep medications was associated with a lower risk of achieving an NCNs score of 7 or higher (RR = 0.68, 95% CI = 0.57-0.83, p < 0.01).

## Discussion

Our study represents a pioneering effort in Japan to quantitatively assess and verify nursing care provided to critically ill patients at discharge using NCNs as a key indicator. This study aimed to assess the NCNs of survivors of COVID-19 and septic shock at discharge or transfer from acute care hospitals and to comparatively examine the physical burden of PICS. Although no statistically significant differences in NCNs were found between the COVID-19 and sepsis survivor groups, both groups exhibited high NCNs levels upon discharge. Furthermore, modified Poisson regression analysis identified prolonged mechanical ventilation, older age, pre-existing cerebrovascular diseases, and in-hospital risky behavior as risk factors for NCNs scores exceeding 7 points at discharge. These findings suggest that COVID-19 may be associated with a higher risk of elevated NCNs compared to other diseases, while sleep medication prescriptions may potentially reduce this risk.

A retrospective observational study conducted in Belgium revealed significantly higher NAS and increased nursing hours among COVID-19 patients admitted to the ICU [11]. However, the control group included only 10% of sepsis patients, indicating a heterogeneous patient population. In our study, by directly comparing COVID-19 and septic shock patients, we found elevated NCNs at discharge or transfer for both groups. While NAS assesses nursing workload and our NCNs assessment evaluates patient condition and nursing care implementation, the scope of these assessments differs. Nevertheless, our quantitative evaluation using the NCNs assessment provides novel insights into nursing care assessments. An Italian observational study reported that nearly half of COVID-19 patients discharged from acute care settings experienced severe impairments in physical function and ADLs [23]. Moreover, a Japanese study using the health-related quality of life measure found that sepsis survivors required assistance with ADLs at discharge [10]. Our findings align with these studies, showing that both COVID-19 and septic shock survivors require high levels of nursing care at discharge, with COVID-19 patients possibly experiencing a longer period of physical impairment compared to other ventilated patients. These results underscore the importance of establishing benchmarks for post-acute care in ICU survivors, particularly in developing and supporting nursing care plans. However, NCNs data do not fully reflect all aspects of nursing care in hospitalized patients. The development of comprehensive tools for evaluating nursing care and further quantitative evaluation and research are essential for advancing nursing in our country. Our study also suggested an association between prolonged mechanical ventilation and increased NCNs. Previous studies have shown that prolonged mechanical ventilation is linked to decreased physical function at discharge, lower discharge-to-home rates, and increased transfers to long-term care facilities [24]. Efforts to facilitate earlier weaning from mechanical ventilation may improve ADL recovery post-extubation [25]. However, further research is needed to determine whether reducing the duration of mechanical ventilation alone is sufficient to decrease NCNs. Additionally, a cohort study using the Therapeutic Intervention Scoring System (TISS-28) found that nursing workload increases with age, particularly in patients aged 44 and older [26]. While the previous study used analysis of variance, we calculated RRs to demonstrate the association between age and NCNs. Older patients aged 80 and above with severe COVID-19 have shown decreased ADLs and higher mortality rates, with many unable to perform ADLs independently at discharge [27]. Given the aging population in Japan, we anticipate that older patients with severe conditions will require a higher level of nursing care. Furthermore, a Brazilian cross-sectional study in a neurosurgical ICU found a significant association between nursing workload, as measured by NAS, and patient severity [28], suggesting a heavier nursing burden for patients with cerebrovascular diseases. Likewise, our finding that patients with cerebrovascular comorbidities at admission were associated with a higher risk of increased NCNs indicates that rehabilitation, neurological assessments, and post-stroke symptom management may require significant nursing time and care.

It is noteworthy that inpatient risky behavior was also associated with an increased risk of higher NCNs. We speculate that these behaviors, often linked to delirium and agitation, may influence nursing care. In contrast, the prescription of sleep medications was associated with a decreased risk of higher NCNs. Given evidence that sleep medication use improves sleep quality, reduces delirium, shortens ICU stay, and decreases mechanical ventilation duration in critically ill patients [29], our findings suggest that sleep medications may contribute to the recovery of critically ill patients.

Our results further suggest that ICU survivors requiring ventilatory support are likely to transition to the rehabilitation phase with high NCNs. As PICS is thought to develop during ICU care, its prevention and management, with a focus on post-discharge care, are critical. Interprofessional collaboration to strengthen the ABCDEF bundle is essential [30], and appropriate sleep bundles are considered important factors in the recovery of critically ill patients.

Limitations

Several limitations should be considered when interpreting our findings. First, the inpatient DPC data used were limited to a single facility in Japan, which may restrict the generalizability of the results due to potential regional or demographic biases and could introduce selection bias and information bias. Given the relatively small sample size, further research involving multiple institutions and comparative analyses is needed. Nonetheless, quantitative studies examining NCNs in Japan are scarce, highlighting the need to accumulate evidence on nursing practices in the country. Second, as the DPC data do not include clinical laboratory data or treatment processes, there may be unmeasured confounding factors. The lack of adjustment for patient clinical assessments or severity limits the interpretation of detailed patient evaluations and statistical analyses. Third, when evaluating NCNs quantitatively, the potential impact of measurement bias must be considered. To minimize measurement bias and ensure reliable assessments, careful selection and standardization of measurement methods, training and management of evaluators, and quality control of data are essential. Besides, as DPC data are used for medical claims, they lack detailed information on patient satisfaction or specific nursing care details. Research using diverse data sources, such as patient-reported outcome data and NAS, is necessary for a more comprehensive understanding.

## Conclusions

Our findings highlight the critical importance of comprehensive assessment and early intervention for critically ill patients, including those with COVID-19 and septic shock, aimed at facilitating ICU liberation. This study represents the first effort in Japan to quantitatively evaluate and validate nursing care at discharge for ICU survivors using NCNs. Our findings offer valuable insights into supporting post-discharge care for ICU survivors and contribute to improving the quality of nursing care.

## Appendices

**Table 4:** Calculation method for the Nursing Care Needs score (Section B). Nursing care needs scoring range; Maximum Score:12 point, Minimum Score:0 point. 1) Transferring, oral hygiene, eating, dressing/undressing, patient condition times nursing care implementation equals score. 2) Turning in bed, compliance with inpatient care and medical orders, risk behaviors; patient condition equals score. 3) 1) score plus 2) score equals total score (evaluation).

Key aspects	Patient condition			Nursing care implementation		Score
	0 points	1 point	2 points	0 points	1 point	
Turning in bed	Independent	Requires support	Unable	-	-	point
Transferring	Independent	Partial assistance	Total assistance	Not	Performed	point
Oral hygiene	Independent	Require assistance	-	Not	Performed	point
Eating	Independent	Partial assistance	Total assistance	Not	Performed	point
Dressing/Undressing	Independent	Partial assistance	Total assistance	Not	Performed	point
Compliance with inpatient care and medical orders	Yes	No	-	-	-	point
Risk behaviors	None	-	Present	-	-	point
						Total score point
